# Simultaneous and independent detection of C9ORF72 alleles with low and high number of GGGGCC repeats using an optimised protocol of Southern blot hybridisation

**DOI:** 10.1186/1750-1326-8-12

**Published:** 2013-04-08

**Authors:** Vladimir L Buchman, Johnathan Cooper-Knock, Natalie Connor-Robson, Adrian Higginbottom, Janine Kirby, Olga D Razinskaya, Natalia Ninkina, Pamela J Shaw

**Affiliations:** 1 School of Biosciences, Cardiff University, Museum Avenue, Cardiff, CF10 3AX, UK; 2 Sheffield Institute for Translational Neuroscience (SITraN), University of Sheffield, 385A Glossop Road, Sheffield, S10 2HQ, UK; 3 Pirogov Russian National Research Medical University, Ostrovitianov str. 1, Moscow, 117997, Russian Federation; 4 Institute of Physiologically Active Compounds of RAS, 1 Severniy Proezd, Chernogolovka, Moscow Region, 142432, Russian Federation

**Keywords:** C9ORF72, Amyotrophic lateral sclerosis, Southern hybridisation

## Abstract

**Background:**

Sizing of GGGGCC hexanucleotide repeat expansions within the *C9ORF72* locus, which account for approximately 10% of all amyotrophic lateral sclerosis (ALS) cases, is urgently required to answer fundamental questions about mechanisms of pathogenesis in this important genetic variant. Currently employed PCR protocols are limited to discrimination between the presence and absence of a modified allele with more than 30 copies of the repeat, while Southern hybridisation-based methods are confounded by the somatic heterogeneity commonly present in blood samples, which might cause false-negative or ambiguous results.

**Results:**

We describe an optimised Southern hybridisation-based protocol that allows confident detection of the presence of a *C9ORF72* repeat expansion alongside independent assessment of its heterogeneity and the number of repeat units. The protocol can be used with either a radiolabeled or non-radiolabeled probe. Using this method we have successfully sized the *C9ORF72* repeat expansion in lymphoblastoid cells, peripheral blood, and post-mortem central nervous system (CNS) tissue from ALS patients. It was also possible to confidently demonstrate the presence of repeat expansion, although of different magnitude, in both *C9ORF72* alleles of the genome of one patient.

**Conclusions:**

The suggested protocol has sufficient advantages to warrant adoption as a standard for Southern blot hybridisation analysis of GGGGCC repeat expansions in the C9ORF72 locus.

## Background

Hexanucleotide repeat expansion in the *C9ORF72* locus has been identified as a genetic cause, or at least a strong risk factor, for a significant proportion of amyotrophic lateral sclerosis cases [1,2]. It is unknown whether the expansion causes neuronal injury through a toxic gain of function, haploinsufficiency or both mechanisms. Recent studies suggested that not a protein encoded by the *ORF72* gene but dipeptide products of expanded repeat region translation might be toxic for neurons [3,4]. Gain of function is consistent with apparently autosomal dominant inheritance, parallels with other neurodegenerative disorders caused by an intronic expansion [5], and the suggestion of anticipation [6]. Difficulty in estimating the size of the *C9ORF72* expansion has precluded investigation of possible correlations between the repeat length and disease characteristics such as age of onset, severity, or speed of progression.

A repeat primed PCR technique quickly and reliably determines whether a pathological *C9ORF72* expansion of >30 repeats is present in a DNA sample, [1,2] but does not allow even approximate quantification of the repeat number because the 100% GC content of the repeat sequence precludes PCR through the region. Conversely, commonly used Southern hybridisation protocols for detecting fragments encompassing the region of expansion in the *C9ORF72* locus often produce false-negative results when DNA extracted from peripheral blood is analysed. This is due to high repeat number heterogeneity in these samples, which leads to the appearance of multiple high molecular mass fragments forming a smear that might be difficult to distinguish from non-specific binding of the hybridisation probe to digested genomic DNA, even when stringent hybridisation/washing conditions are employed [7-9]. Here we describe a reproducible protocol for unambiguous detection and sizing of the *C9ORF72* repeat expansion by Southern hybridisation.

## Results and discussion

**Figure 1:**
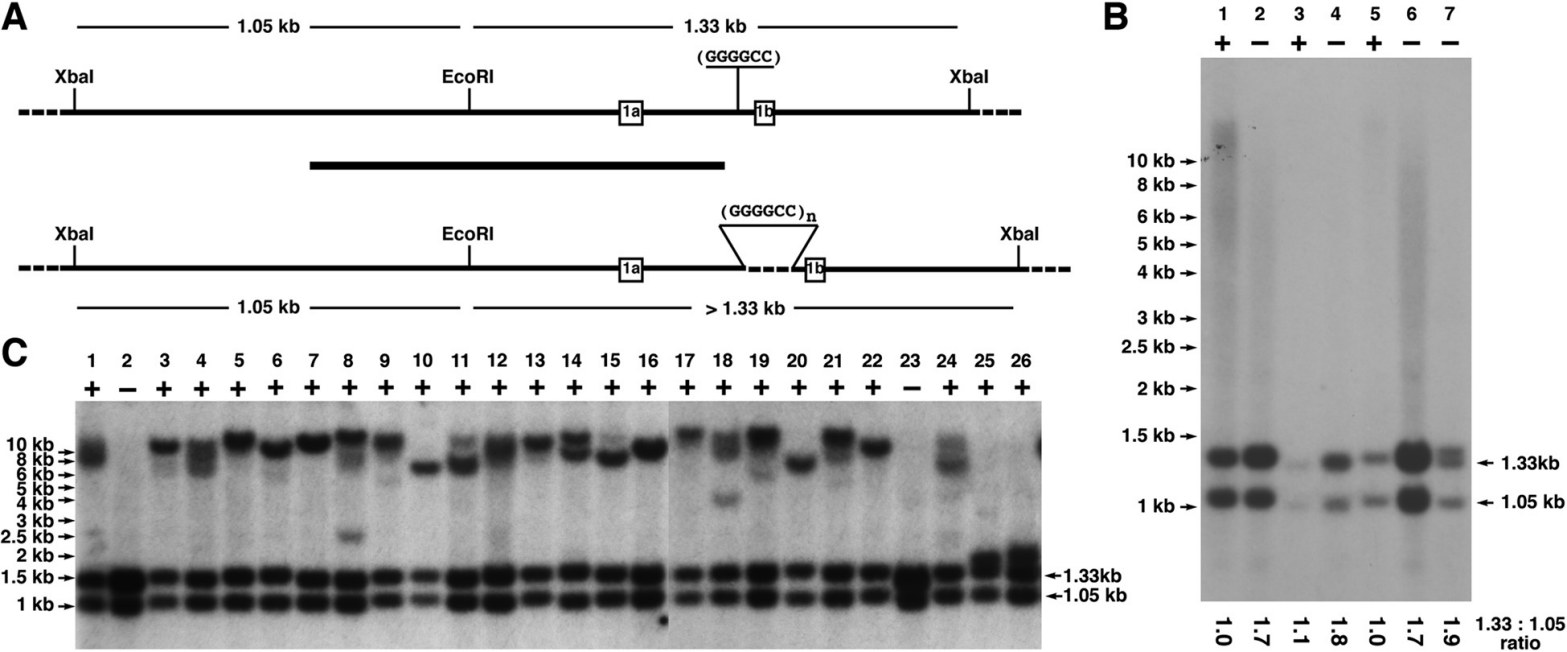
**Southern blot analysis of GGGGCC repeat expansion in the *C9ORF72 *locus. **(**A**) Scheme of a region around exons 1a and 1b of human *C9ORF72* gene with a single copy of GGGGCC sequence (top) or expansion of this repeat (bottom). The black bar in the middle denotes a DNA probe used for Southern blot hybridisation. The sizes of fragments produced by double EcoRI and XbaI digestion and detected by hybridisation with this probe are shown. (**B**) Hybridisation of EcoRI and XbaI digested DNA extracted from the whole blood of patients positive (+) or negative (-) for GGGGCC repeat expansion according to results of the repeat-PCR analysis. The ratios of band intensities obtained by scanning the X-ray film are shown below the image. Note that for sample 7 the intensity used for calculation is combined intensities of two close ~1.33 kb size bands. (**C**) Hybridisation of EcoRI and XbaI digested DNA extracted from the cultured lymphoblastoid cell lines of patients positive (+) or negative (-) for GGGGCC repeat expansion according to results of the repeat-PCR analysis.

Our protocol produces an internal standard band on Southern blots, the size and intensity of which is independent of heterogeneity in the repeat expansion. This is achieved by using as a hybridisation probe a cloned genomic fragment encompassing an internal EcoRI site located close to the repeat expansion region and digesting genomic DNA with two enzymes, EcoRI and XbaI (Figure 1A). The EcoRI site splits the probe into two unequal fragments. Products of a labeling reaction originating from the shorter fragment of the probe hybridise with a 1.05 kb XbaI-EcoRI genomic fragment that does not include the repeat region, while those originating from the longer fragment (~2/3 of the probe length) hybridise with a 1.33 kb XbaI-EcoRI fragment derived from a non-expanded locus, or a larger fragment from a locus with the repeat expansion (Figure 1A).

### Subjects without a pathological C9ORF72 hexanucleotide expansion

Because the target XbaI-EcoRI fragments are quite similar in size (thus, no effect of the transfer efficiency during capillary blotting) but the ratio of probe length for detecting each fragment is approximately 1:2, the ratio of intensity of the two bands in the absence of an expanded allele, should be approximately 1:2. Indeed DNA samples from peripheral blood (Figure 1B, lanes 2, 4, 6) or lymphoblastoid cell lines (Figure 1C, lanes 2, 23 and Additional file 1: Table S1) from ALS patients without the repeat expansion show this pattern.

A swing of the intensity ratio of the bands to approximately 1:1 is an indicator of heterozygosity in the *C9ORF72* locus. If the difference in the number of repeats in the two alleles is small (i.e. no pathological repeat expansion in either allele) two close and equally weighted bands are present on Southern blots even when whole blood cell DNA is analysed, as illustrated in Figure 1B lane 7, using a DNA sample from a patient carrying one allele with a single copy of the GGGGCC sequence and another allele with 15 copies.

### Subjects with a pathological C9ORF72 hexanucleotide expansion in one allele

**Figure 2:**
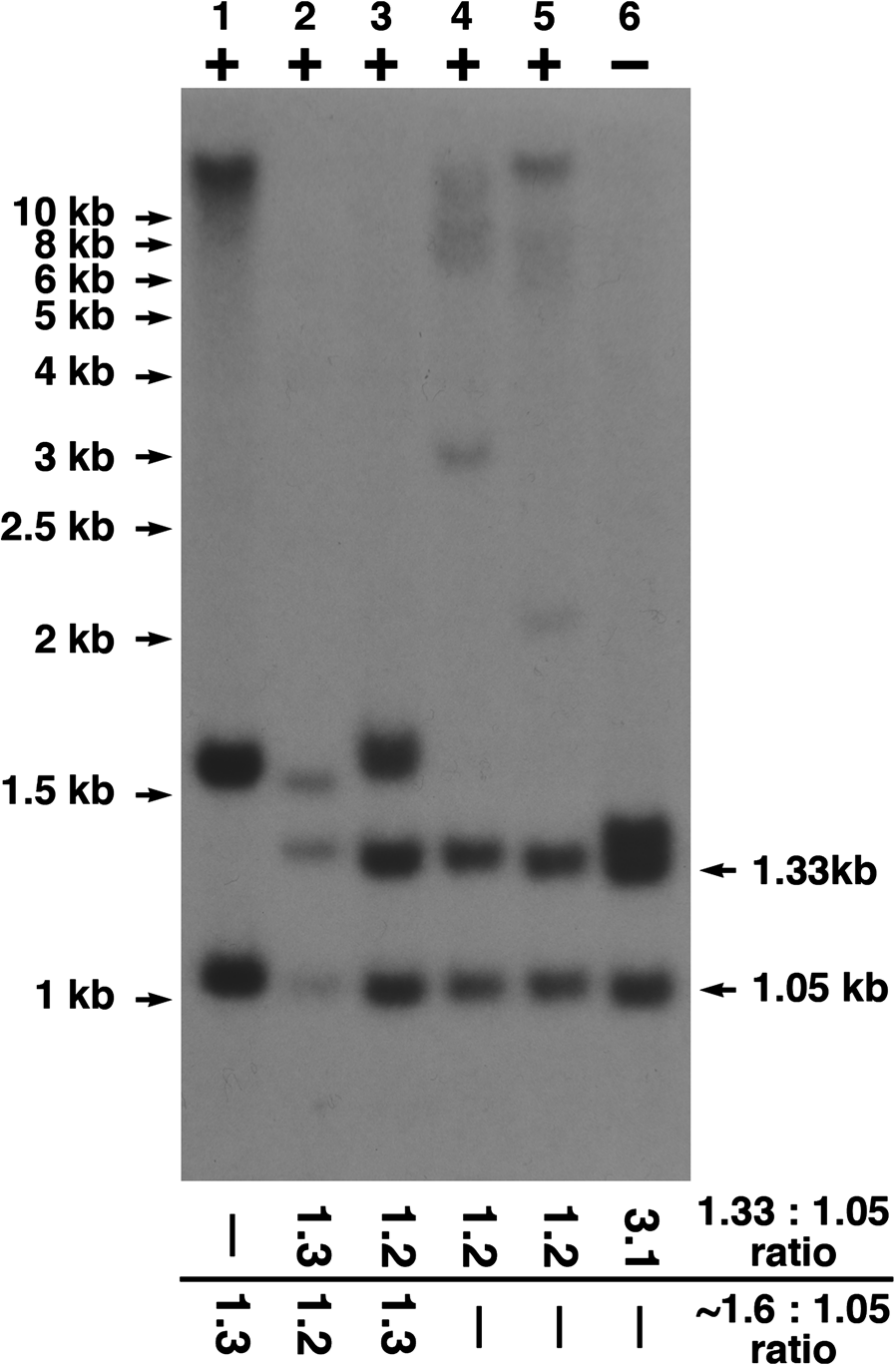
**Identification of a patient with GGGGCC repeat expansion in both alleles of the *C9ORF72 *locus. **Hybridisation of EcoRI and XbaI digested DNA extracted from the cultured lymphoblastoid cell lines of patients positive (+) or negative (-) for GGGGCC repeat expansion according to results of the repeat-PCR analysis. Note the presence of ~1.6 kb band representing an allele with relatively small, although what is considered pathological, i.e. >30, number of repeats in one of the *C9ORF72* loci in the genome of three patients (lanes 1 – 3). In contrast to patients carrying the second allele without pathological repeat expansion (lanes 2–6), one patient (lane 1) has the second allele with very high number of repeats. Where relevant, the ratios of the 1.33 kb to 1.05 kb and/or ~1.6 kb to 1.05 kb bands intensities are shown below the image. Note that for sample 6 the intensity used for calculation is combined intensities of two close ~1.33 kb size bands.

In cases with a pathological repeat expansion in one allele, a band of larger size is detected in DNA samples extracted from patients’ lymphoblastoid cell lines. Examples of these can be found in Figure 1C lanes 10 and 20 (~6 kb band corresponds to an allele with ~750 repeats), 15 (~7.5 kb band corresponds to an allele with ~1000 repeats), 16 and 22 (~8.5 kb band corresponds to an allele with ~1200 repeats), 6 (~10 kb band corresponds to an allele with ~1500 repeats), 3, 7 and 13 (~12 kb band corresponds to an allele with ~1800 repeats), 5, 8, 17 (>12 kb band corresponds to an allele with >2000 repeats). For accuracy of size estimates see Methods section. The presence in the sample of more than one of these larger bands (for example, lanes 4, 8, 14, 18 in Figure 1C, lanes 4, 5 in Figure 2) might indicate instability of the GGGGCC repeat region in the cultured lymphoblastoid cells. Alternatively it might reflect instability and somatic heterogeneity of the repeat region in ALS patients’ lymphoid cells, coupled with non-monoclonal origin of the analysed lymphoblastoid cell lines. In whole blood DNA such somatic heterogeneity is clearly evident in some samples, leading to the appearance of multiple bands that coalesce into an ambiguous smear on Southern blots (Figure 1B, lanes 1, 5). For two other samples in the same panel (Figure 1B, lanes 2, 6) lower quality of DNA caused appearance of similar smears but the ratio of 1.05 kb and 1.33 kb band intensities in these cases are close to 1:2 suggesting that both alleles of the C9ORF72 locus have small number of GGGGCC repeats. Therefore, even in those cases with an ambiguous smear, the presence of a pathological allele could be immediately predicted from the ~1:1 ratio of 1.05 kb and 1.33 kb band intensities.

### Subjects with a pathological C9ORF72 hexanucleotide expansion in both alleles

The described protocol readily identifies patients with repeat expansions within both *C9ORF72* loci. These cases are characterised by the absence of a 1.33 kb band on Southern blots, as illustrated in Figure 2, lane 1 for a patient carrying one allele with ~50 and another allele with >2000 GGGGCC repeats in the *C9ORF72* locus.

### Southern hybridization in different tissues from the same subject

**Figure 3:**
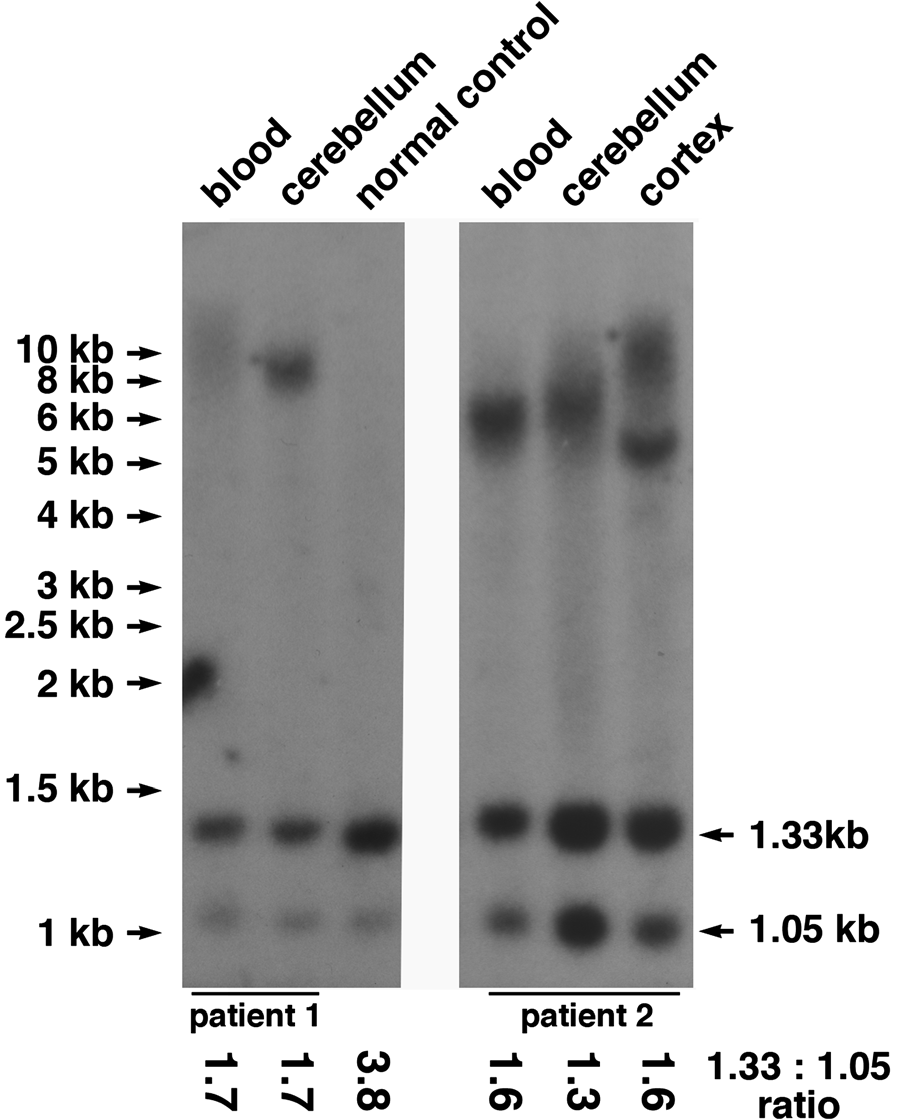
**Somatic cell genomes of ALS patients have variable number of GGGGCC repeats in the *C9ORF72 *locus. **Two sections of the same Southern blot (see raw image in Additional file 2: Figure S1) show hybridisation of EcoRI and XbaI digested DNA extracted from peripheral blood, cerebellum and cerebral cortex of two patients positive for GGGGCC repeat expansion. The ratios of the 1.33 kb to 1.05 kb bands intensities are shown below the image. Note that in this case these ratios are unconventionally high, which is due to the gel has been run for too long and therefore the 1.05 kb bands appeared too close to the edge of the gel, resulting in less efficient transfer and hybridisation of this band. However, the ratio for normal DNA sample is still approximately twice as high as the ratio for all DNA samples with GGGGCC repeat expansion.

When our Southern hybridisation protocol was used for analysis of genomic DNA extracted from peripheral blood, cerebellum, cortex and a lymphoblastoid cell line of patients with GGGGCC repeat expansion, a different pattern of high molecular mass fragments has been detected for each DNA sample of the same patient, as illustrated in Figure 3 and Additional file 2: Figure S1. This observation strongly suggests that an expanded repeat region in the *C9ORF72* locus is unstable not only in nucleated blood cells but also in other somatic cells, including cells of the central nervous system.

Although a ^32^P-radiolabeled hybridisation probe remains the best option for detection of GGGGCC repeat expansions in the *C9ORF72* locus with the generation of clearer and more quantitative results compared to those obtained using DIG-labeled probes, in a pilot experiments we successfully used the latter detection method with our protocol (data not shown).

## Conclusions

Our optimised protocol for Southern hybridisation allows simple and confident detection, as well as sizing of the repeat expansion in various types of cells and tissues. Thus we recommend this protocol as an acceptable standard for Southern blot hybridisation analysis of GGGGCC repeat expansions in the *C9ORF72* locus.

## Methods

### DNA extraction

Whole blood samples were obtained from the Sheffield MND Blood DNA Biobank and CNS tissue was obtained from the Sheffield Brain Tissue Bank. The South Sheffield Research Ethics Committee approved the study, and informed consent was obtained for all samples. Lymphoblastoid cell lines were obtained from patients with ALS from the Wellcome Trust/Motor Neurone Disease Association-funded ALS/MND DNA bank and associated lymphoblastoid cell line repository in the UK.

Before lysis with a digest buffer (50 mM Tris–HCl pH 8.0; 100 mM NaCl; 5 mM EDTA; 1% SDS; 2 mg/ml proteinase K) frozen CNS tissues were grinded in liquid nitrogen. Following overnight digestion at 55°C genomic DNA was extracted twice with phenol and once with phenol/chloroform and ethanol precipitated.

### Southern hybridisation

A genomic fragment was amplified from a human DNA using oligonucleotide primers AGTTCCAGAGCTTGCTACAG and GAACAGTAGGAAAAGGGTCTG and cloned into the pCR-BluntII-TOPO vector (Invitrogen) to produce a pCh9.1 plasmid carrying an insert used as the hybridisation probe (Additional file 3: Figure S2). Well-established and widely used methods of Southern transfer, preparation of the probe and hybridisation procedure were used. In brief, approximately 20 μg of genomic DNA was digested with EcoRI and XbaI and the resulting fragments separated in 1% TAE agarose gel. After incubating the gel in 0.25 N HCl for 20 min, 0.5 N NaOH; 1.5 M NaCl for 40 min and 0.5 M Tris–HCl pH7.2; 3 M NaCl for 40 min at room temperature, DNA was transferred to a nylon membrane (Hybond N+, GE Healthcare) by capillary blotting [10]. An eukaryotic insert of the plasmid pCh9.1 was excised using KpnI and XbaI, separated from the plasmid backbone in agarose gel, purified using Qiagen kit and used for preparing hybridisation probes. The probe was labelled with ^32^P in a nick-translation reaction [11]. Blots were prehybridised in HB (4×SSC; 0.5% SDS; 5×Denhardt’s solution; 100 μg/ml denatured salmon testis DNA) at 67°C for 4 h. Labelled DNA was denatured by incubation at 100°C for 5 min followed by immediate mixing with ice–cold HB. Hybridisation was carried out at 67°C for 16–20 h. Blots were washed 3 times in in 2×SSC; 0.2% SDS at 67°C and exposed to X-ray film. The detailed protocol as well as the pCh9.1 plasmid carrying an insert used as the hybridisation probe can be obtained by sending a request to Vladimir Buchman (buchmanvl@cf.ac.uk).

DNA extracted from 32 lymphoblastoid cell lines obtained from the MND National Biobank was used for Southern hybridisation analysis. This included 29 cases with a *C9ORF72* expansion identified by repeat-PCR and 3 cases with non-*C9ORF72* ALS. For certain cases DNA was also extracted from peripheral blood and CNS tissue available from the Sheffield Brain Tissue and DNA bank.

### Quantification of the band intensity ratios

When at least one normal (without repeat expansion) DNA sample is present on the blot as a reference, the difference in relative intensity of 1.33 kb and 1.05 kb bands in this sample and in samples with repeat expansion can be easily recognised by eye. However, this difference can be quantified by scanning X-ray films, measuring bands using a software available for every gel documentation system and calculating the ratio of pixels in 1.33 kb and 1.05 bands for each sample. Even when for any technical reasons, the ratio of bands in samples with repeat expansion is higher than conventional 1:1, this is accompanied by corresponding increase of the ratio in a normal sample(s) present on the same Southern blot, which still allows discrimination of repeat-bearing and normal cases by eye. An example of this is shown in Figure 3.

### Estimation of the number of repeats within an expanded allele

The number of repeats within the expanded allele is estimated based on the size of corresponding fragment in base pairs minus the size of the non-expanded fragment (1330 base pairs) and divided by the size of the repeat unit (6 base pairs). For example, if the size of a fragment was 3 kb, estimated number of repeats in the corresponding locus is (3000 – 1330)/6 = 278. As with any method based on agarose gel electrophoresis, an accuracy of the repeat number estimate using the described protocol varies with the size of detected fragments. In the range of the repeat-bearing fragment size between 1.5 kb (which corresponds to an upper limit for a “normal” allele, i.e. less than 30 repeats = 0.18 kb + 1.33 kb -> 1.51 kb) and 3 kb (~300 repeats) the accuracy of the estimate might be around 10 repeats; between 3 kb and 6 kb (~750 repeats) it drops to ~50 repeats and between 6 kb and 10 kb (~1500 repeats) – to 200 repeats. Any bands above 12 kb should be considered as >2000 repeats.

## Abbreviations

ALS: Amyotrophic lateral sclerosis; CNS: Central nervous system; DIG: Digoxigenin.

## Competing interests

The author(s) declare that they have no competing interests.

## Authors’ contributions

VB and NN designed the Southern hybridisation probe and conceived the protocol. JCK, JK and PJS participated in the design of the study. VB, JCK, NN, AH, ODR and NCR carried out experiments. VB, NN, JCK and PJS participated in data analysis. VB, JCK and PJS drafted the manuscript. All authors read and approved the final manuscript.

## Supplementary Material

Additional file 1: Table S1 Ratios of 1.33 kb and 1.05 kb bands intensities for samples shown in Figure 1C. Click here for file

Additional file 2: Figure S1 Raw image of the Southern blot used for preparing main Figure 3. Click here for file

Additional file 3: Figure S2 Scheme of a DNA fragment used as a hybridisation probe inserted into a polylinker cloning site of pCR-Blunt II-TOPO plasmid. The sequence of the plasmid polylinker region is from the Invitrogen manual for TOPO cloning kit. Click here for file
