# Supplementary material for: Simultaneous and independent detection of C9ORF72 alleles with low and high number of GGGGCC repeats using an optimised protocol of Southern blot hybridisation
Source: Mol Neurodegener. 2013 Apr 8;8:12. doi: 10.1186/1750-1326-8-12 (PMC3626718; doi:10.1186/1750-1326-8-12)
Supplement: Additional file 1: Table S1 — Ratios of 1.33 kb and 1.05 kb bands intensities for samples shown in Figure 1C. [file 1750-1326-8-12-S1.pdf]

Supplementary Table 1. Ratios of 1.33 kb and 1.05 kb bands intensities for samples shown in Figure 1C

	1	2	3	4	5	6	7	8	9	10	11	12	13	14	15	16	17	18	19	20	21	22	23	24	25	26
GGGGCC repeats	+	-	+	+	+	+	+	+	+	+	+	+	+	+	+	+	+	+	+	+	+	+	-	+	+	+
1.33 :1.05 ratio	1.1	2.1	1.1	1.0	1.0	1.1	1.0	1.0	0.9	1.0	1.0	1.0	1.1	1.0	1.0	1.0	1.1	1.1	1.0	1.0	1.1	1.2	2.2	1.1	1.1	1.1
