# Supplementary figures and images for: Simultaneous and independent detection of C9ORF72 alleles with low and high number of GGGGCC repeats using an optimised protocol of Southern blot hybridisation
Source: Mol Neurodegener. 2013 Apr 8;8:12. doi: 10.1186/1750-1326-8-12 (PMC3626718; doi:10.1186/1750-1326-8-12)

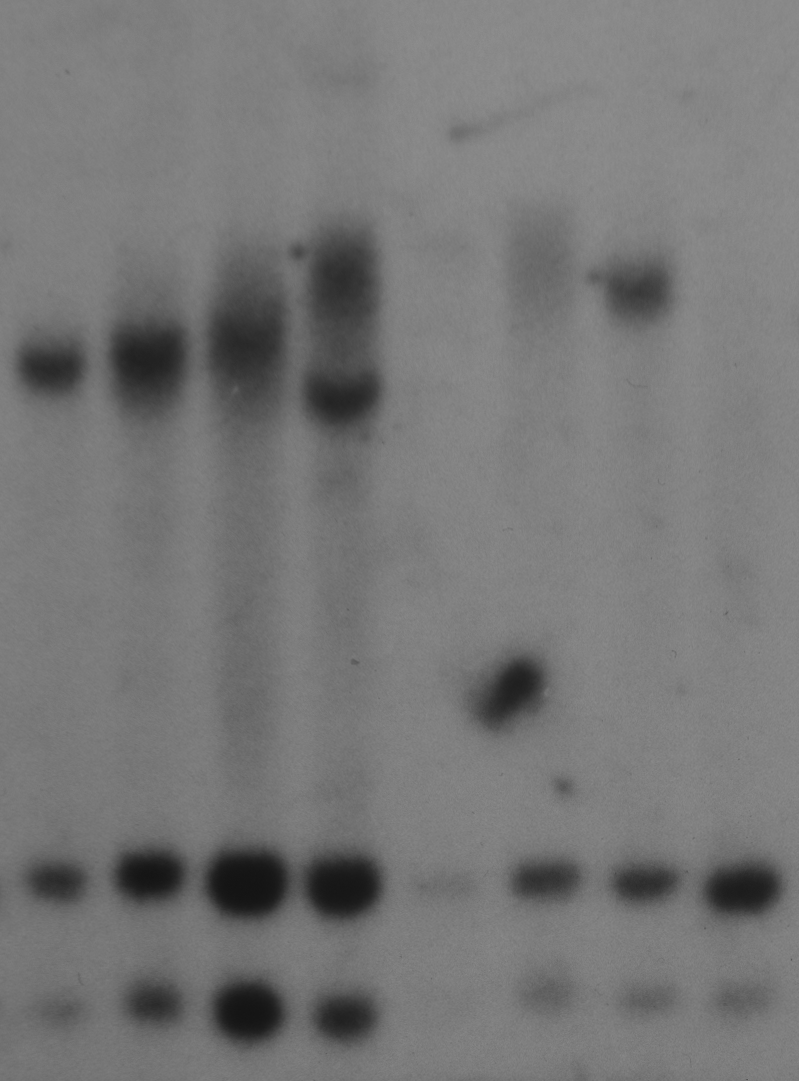

Supplement: Additional file 2: Figure S1 — Raw image of the Southern blot used for preparing main Figure 3. [file 1750-1326-8-12-S2.tiff]
