# Supplementary material for: Simultaneous and independent detection of C9ORF72 alleles with low and high number of GGGGCC repeats using an optimised protocol of Southern blot hybridisation
Source: Mol Neurodegener. 2013 Apr 8;8:12. doi: 10.1186/1750-1326-8-12 (PMC3626718; doi:10.1186/1750-1326-8-12)
Supplement: Additional file 3: Figure S2 — Scheme of a DNA fragment used as a hybridisation probe inserted into a polylinker cloning site of pCR-Blunt II-TOPO plasmid. The sequence of the plasmid polylinker region is from the Invitrogen manual for TOPO cloning kit. [file 1750-1326-8-12-S3.pdf]

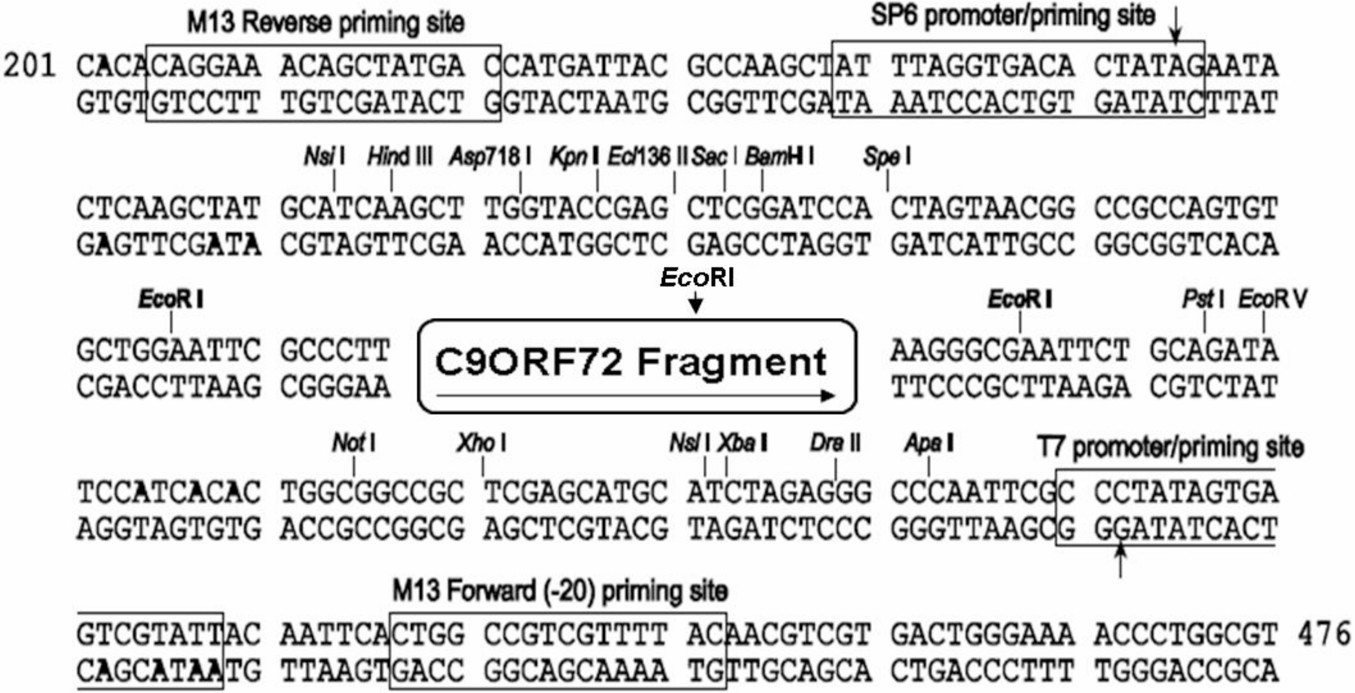
M13 Reverse priming site
SP6 promoter/priming site
201 CACACAGGAA ACAGCTATGA CCATGATTAC GCCAAGCTAT TTAGGTGACA CTATAGAATA
GTGTGTCCTT TGTCGATACT GGTACTAATG CGGTTCGATA AATCCACTGT GATATCTTAT
Nsi I
Hind III
Asp718 I
Kpn I
Ecl136 II
Sac I
BamH I
Spe I
CTCAAGCTAT GCATCAAGCT TGGTACCGAG CTCGGATCCA CTAGTAACGG CCGCCAGTGT
GAGTTCGATA CGTAGTTCGA ACCATGGCTC GAGCCTAGGT GATCATTGCC GGCGGTCACA
EcoR I
EcoRI
EcoR I
Pst I
EcoR V
GCTGGAATTC GCCCTT
CGACCTTAAG CGGGAA
C9ORF72 Fragment
AAGGGCGAATTCT GCAGATA
TTCCCGCTTAAGA CGTCTAT
Not I
Xho I
Nsi I
Xba I
Dra II
Apa I
T7 promoter/priming site
TCCATCACAC TGGCGGCCGC TCGAGCATGC ATCTAGAGGG CCCAATTCGC CCTATAGTGA
AGGTAGTGTG ACCGCCGGCG AGCTCGTACG TAGATCTCCC GGGTTAAGCG GGATATCACT
M13 Forward (-20) priming site
GTCGTATTAC AATTCACTGG CCGTCGTTTT ACAACGTCGT GACTGGAAAA ACCCTGGCGT 476
CAGCATAATG TTAAGTGACC GGCAGCAAAA TGTTGCAGCA CTGACCCTTT TGGGACCGCA
